# Dual-Purpose Water Buffalo Production Systems in Tropical Latin America: Bases for a Sustainable Model

**DOI:** 10.3390/ani11102910

**Published:** 2021-10-08

**Authors:** Aldo Bertoni, Adolfo Álvarez-Macías, Daniel Mota-Rojas, José Luis Dávalos, Antonio Humberto Hamad Minervino

**Affiliations:** 1 Master’s Program in Agricultural and Livestock Sciences [Maestría en Ciencias Agropecuarias], Universidad Autónoma Metropolitana (UAM), Xochimilco Campus, Mexico City 04960, Mexico; aldo_bm@hotmail.com; 2 Neurophysiology, Behavior, and Animal Welfare Assessment, Department of Animal Production and Agriculture (DPAA), Universidad Autónoma Metropolitana (UAM), Xochimilco Campus, Mexico City 04960, Mexico; 3 Department of Economics, Administration and Rural Development, Universidad Nacional Autónoma de Mexico (UNAM), Mexico City 04510, Mexico; jldf@fmvz.unam.mx; 4 Laboratory of Animal Health, LARSANA, Federal University of Western Pará, UFOPA, Rua Vera Paz, s/n, Santarém 68040-255, PA, Brazil; ah.minervino@gmail.com

**Keywords:** agrosilvopastoral, dual purpose systems, integral management, river buffalo, tropics

## Abstract

**Simple Summary:**

Buffaloes in the Latin American tropics have gained relevance in recent years thanks to the rusticity and productivity of this species and the potential of these ecosystems. Through a specialized literature review, the options were explored to promote efficient, profitable and sustainable dual-purpose production systems, which implied taking into consideration the characteristics of a fragile environment such as the tropics but with abundant forage resources. Buffaloes have distinctive characteristics with respect to cattle, such as a high fat milk content, ability to produce healthy and heavy calves from dairy cows and their need for thermoregulation, which implies the availability of water ponds and shade in grazing areas. Therefore, we propose the basis for designing intensive rotational grazing models and, under certain silvopastoral conditions, taking advantage of the gregarious behavior of buffaloes. These models require integral forms of management, including the organization and participation of producers in commercial channels, in order to value meat, milk and dairy products, given that their nutritional qualities allow them to be sold as differentiated products. There have been successful experiences in the region with this dual-purpose model, which, with the necessary adaptations, can be strengthened with buffaloes.

**Abstract:**

Tropical regions of Latin America have been incorporated into development in recent decades, with extensive cattle ranching as one of the main economic activities but without adequate planning, drastically degrading the ecosystem. In recent years, buffalo production has been incorporated into the region, with possibilities for development in profitable and sustainable models. To study this option in depth, a broad bibliographic review was carried out focusing on the ecological characteristics of tropical zones and the physiological and productive characteristics of buffaloes. We also investigated the structure and functioning of dual-purpose systems that have worked in cattle and that can be optimized with this alternative animal species. The possibility of taking sustainable advantage of abundant forage resources in the region was detected through intensive grazing models, as well as agrosilvopastoral systems, due to the gregarious qualities of buffaloes and responding to their thermoregulation needs. In this way, the productive and regenerative capacities of the dual-purpose system could be increased, as well as the quality of meat and milk, which could be marketed as differentiated products, taking advantage of their outstanding nutritional qualities. Integral management of the dual-purpose system is proposed, retaking the bases of the original model of family characters, diversified and with low investments and risks, which with specific innovations can be an effective development option for producers in the region.

## 1. Introduction

Tropical regions are characterized by diverse and abundant biodiversity but also by fragility under inadequate systems of territorial management. In Latin America, these ecosystems are among the last remaining areas to be colonized, but optimizing this opportunity for productive exploitation requires a profound knowledge and understanding of their structure and functioning to help minimize the imbalance that anthropogenic activities trigger [1]. Sadly, a retrospective view confirms that this has not occurred but that the regional economic development has entailed high costs, especially in social and ecological terms, because the operations have occupied areas populated by vulnerable social groups and caused the degradation of one of the most complex ecosystems on Earth: tropical jungles. Climate change has aggravated this problem, because the deterioration of natural resources leaves it especially vulnerable to changes in rainfall patterns and has resulted in higher temperatures and a more frequent incidence of natural phenomena like hurricanes and drought [2].

One predominant economic activity in the tropics is livestock-raising, which encountered an adequate environment for development, especially from the second half of the 20th century to the present. There is, however, evidence that the ecosystems of regions like the Amazon have succumbed to the clearing of forested areas to open up pastures and, more recently, for crop production. One unfortunate feature of the expansion of cattle-ranching has been the lack of planning, despite the emission of laws and policies designed to foment sustainable development. This phenomenon has been observed in Brazil and Mexico [3]. Cattle have been figured as the key protagonists in this development process and led to the elaboration of a dual-purpose model designed to obtain meat and dairy products simultaneously [4]. This flexible system seeks to guarantee producers steady sources of income while increasing the supply of meat and milk products to sustain processes of industrial development and urbanization in almost all countries in the region.

The insertion of the dual-purpose livestock production system into numerous countries in the region requires installing the typical equipment and infrastructure that support the meat and dairy industries, but it has generated employment and a substantial economic return. In recent years, the essential elements of the dual-purpose system have been transferred to operations that are breeding water buffaloes (*Bubalus bubalis*) in tropical areas of Latin America. Although the majority of water buffalo herds, estimated as more than 208 million heads, are raised in Asia, the American continent has more than 2.5 million buffalo, with the vast majority being raised in the Latin America tropical zone [5]. This industry is gaining strength thanks to the rusticity and versatility of this species, especially in zones marked by high humidity [6], such as Brazilian Amazon wetlands and large areas of Venezuela [7]. The river buffalo has gradually demonstrated its productive efficiency and capacity to adapt to the environmental and behavioral exigencies of tropical climates, while appreciation of the nutritional value of its meat and dairy products is growing [8,9,10,11,12,13,14,15].

Ensuring the viability of raising water buffaloes in the tropics requires, on the one hand, evaluating the key characteristics of the region, including the soil types, temperature, precipitation patterns and the wealth of forage resources, emphasizing the endemic varieties. On the other, it is necessary to deepen the knowledge of the characteristics of the water buffalo as a species so as to better attend to its requirements for basic comfort, such as the availability of ponds and/or shade to facilitate the process of thermoregulation, for example, and, thus, allow them to realize their full productive potential [16]. Fostering a sustainable model demand managing resources rationally and supporting soil–plant–animal interactions and their feedback processes to minimize the impact on natural resources and greenhouse gas emissions. One specific recommendation is to implement a strict planning process for managing forage resources that takes full advantage of the different strata of vegetation, from herbaceous to arboreal species, including shrubbery [2]. It is also necessary to ensure that the systems are well-managed so they can generate sufficient incomes for producers by ensuring access to markets that offer attractive prices, recognize the value of high-quality buffalo milk and meat products and compensate and/or incentivize ranchers’ efforts to gradually reconvert, technologically, their productive units [6].

Against this background, the present article discusses elements for understanding the importance and dynamics of tropical areas in Latin America by focusing on their physico-biotic characteristics, options for developing production systems for the water buffalo and the incorporation of technologies that support the principles of sustainability and allow producers and their families to enhance their quality of life through the increase of a financial income due to water buffalo production. This study begins with a broad literature review that allows us to characterize the tropical zones, the dual-purpose system and its associated management schemes and the eventual organization of alternative commercial channels. Overall, our analysis brings advances to the way in which sustainable production models for water buffaloes are conceived.

## 2. The Tropics: A Privileged Region for the Development of Water Buffaloes

Tropical regions are characterized by a vast biodiversity that provides principally abundant vegetable and hydric resources. Due to the high productive potential of these ecosystems, numerous initiatives have been launched, often with official support, to exploit them and transform them into sources of economic wealth. Livestock-raising has been one of the principal productive options explored, but the complexity of these ecosystems has not always been fully taken into account when it comes to elaborating sustainable management schemes that ensure durability. As a result, significant obstacles to livestock development have surfaced to limit the productivity while also contributing to the dramatic degradation of broad areas of native biomes, such as forests. Specifically, it has not been possible to generate, and then transfer to producers, technological models appropriate for the diversity of tropical environments. Instead, the tendency has been to attempt to adapt technological models from temperate zones, despite the fact that they have been exposed as inefficient and inadequate when it comes to stabilizing and regenerating natural resources or mitigating the effects of climate change [17,18]. 

The predominant types of livestock development in the tropics are extensive. They seek to take advantage of the region’s exuberant vegetation and, above all, the variety of forage resources. With the notable expansion of cattle ranching, enormous pressure has been exerted on the forest areas, which have been drastically reduced in size, as they have been cleared to make way for pastures. Cattle ranching is not the only culprit in these processes, as it is important to recognize that other activities and events have had a similar effect, such as agriculture, urbanization, mining, oil exploitation and fires, which have manifestly violated these ecosystems [2,3]. 

For buffaloes to fully develop their productive potential in tropical ecosystems without excessively altering the ecological balance of an area, it is essential to know the main characteristics of this species, to develop efficient management for the productive systems and, at the same time, mitigate, for example, the relative seasonal scarcity of forage plants (and their low nutritional quality) due to the effects of drought, flooding and cyclones [19]. In what follows, the basic elements of the physical and biotic factors of these regions and the richness of their forage resources are discussed.

### 2.1. Physico-Biotic Aspects of the Tropics

Tropical regions are located between the tropics of Cancer and Capricorn, which means that they have distinctive characteristics in relation to latitude, altitude, temperature and precipitation [20]. The tropics lie between the equator and 25° latitude in the Northern and Southern Hemispheres and are predominantly in coastal areas, where they are largely at the sea level. This means, for example, that sunlight reaches the region directly, so the temperatures throughout the year are comparatively high and do not vary greatly. These conditions constitute one of the main triggers for the high photosynthetic capacity of vegetation in these regions that produces abundant biomass [21]. This geographic location also favors humidity currents that frequently sweep these areas, with precipitation rates that often generally range between 2000 and 3000 mm and can exceed 4000 mm in areas of higher precipitation. The relative humidity ranges from 77 to 88% [20,21], and the average temperatures in tropical regions remain between 24 and 27 °C throughout the year, with small fluctuations from month to month or year to year [22]. These predominantly warm conditions stimulate the formation of cumulus and cumulonimbus clouds that produce frequent thunderstorms [20].

Two broad tropical areas can be identified. The first is the so-called dry tropic zone characterized by the Aw climate in Köppen’s classification; that is, hot subhumid with summer rains. The second is the tropical humid climate (Af) distinguished as hot and humid with year-round rains [20]. The potential for raising water buffaloes discussed herein centers on regions with the latter climate. In the case of Mexico, this region stretches from Southern Veracruz to Campeche, passing through Tabasco and including part of Northern Chiapas. This climate is also characteristic of broad zones of Central America, such as Costa Rica, Panama and, further south, ample portions of Brazil, Colombia, Venezuela, Ecuador, Peru and Bolivia, primarily [23,24].

In terms of soil, tropical areas have relatively thin layers due to the accelerated circulation of nutrients between soil and vegetation that, through their rapid metabolism, absorbs large amounts of the nutrients from the substrate, later reintegrating them through the abundant dead vegetable matter that accumulates as organic materials. For this reason, the soils are originally very fertile, so, in the first years after forest clearing, they produce high yields of forage plants and agricultural crops. These levels gradually decrease, however, especially if the fields are not fertilized in some way, since cultivation interrupts the accelerated growth cycle characteristics of the natural vegetation [25]. Another important aspect of these soils is that they are rich in clay and organic materials, so they tend to retain high levels of moisture. During the rainy season, they tend to form marshy areas that, in extreme cases, become seasonal bodies of water [21]. It is in these zones that river buffaloes find an adequate environment for supporting their development, because they utilize those ponds to regulate their body temperature [26], as has been demonstrated in Argentina, Costa Rica and Colombia, and is documented below [9,23].

The geographical and environmental circumstances of the tropics discussed above frequently produce a natural vegetation of the forest type, with high evergreen vegetation in the humid zones and medium and low sub-deciduous and deciduous covers in drier areas [20]. A broad range of grasslands have developed in flat and hilly zones where anthropogenic impacts have led to invasions of species of African origin, like the Star of Africa, Guinea and Pangola grasses. Due to their stoloniferous growth pattern and because they are classified as C4 grasses—that is, plants capable of carrying out photosynthesis even in environments with high temperatures and intense solar radiation—they provide pastures with large biomass and high yields of green material per hectare, though their yields of dry material and bromatological value are only medium-to-low [25].

In summary, compared to temperate zones, the humid tropics are characterized by greater contributions of energy in the form of vapor and water flows, more intense precipitation, the rapid weathering of inorganic and organic materials and the reception of large volumes of water and sediment. Water currents and the amounts of solids and organic carbon also show proportionally greater rates and magnitudes [20]. These factors combine to produce the abundant supplies of forage—described in the following section—that have functioned as one of the main incentives for expanding and consolidating cattle ranching in the tropics, where buffaloes stand out as a species with great potential, since they have gradually demonstrated their capacity to adapt to these environments [14]. For this assemblage to function, however, it is essential to undertake the adequate planning of production systems and forms of development to ensure that they harmonize with the physico-biotic and geographical characteristics of these ecosystems [27,28].

### 2.2. The Productive Potential of Forage Resources in Tropical Zones

Studies in the tropics have identified a broad variety of forage species. Indeed, the list compiled continues to grow, because new varieties, both national and imported, have been introduced and progressively increased their productive potential. In fact, the predominance of the native grasses and those of African origin that persist today in ample areas of the region have been complemented by the incorporation of new species, such as the genera *Brachiaria* and *Pennisetum*, which enhance forage productivities and tend to produce greater yields on ranches, especially when included in the rotating pasture systems that have been implemented more frequently in recent years [18]. We must recognize, however, that ample sectors of producers maintain traditional continuous pasturing systems or move their cattle between the rainy and dry seasons in search of better base forage [19]. One important example is found in Pará State, Brazil, in the Lower Amazon and Marajó regions, where the livestock breeding system is dependent on natural and communal grasslands that grow in the floodplains, with the seasonal alternations between the high and low water levels shaping the livestock herd management [29].

Grasses like African stargrass (*Cynodon plectostachyus*), Guinea (*Panicum maximum*), Aleman (*Echinochloa polystachya*) and Bermuda (*Cynodon dactylon*) have expanded notably since the 1960s, while other species have gradually been identified and improved. These include some of the genus *Brachiaria*, such as Pará (*B. mutica*), Chetumal (*B. humdicola*) and, more recently, Mulato (*B. hibrido*). Other species found are Llanero (*Andropogon gayanus*), Buffel (*Cenchrus ciliaris*), Pennisetum and Taiwan (*P. purpureum*), the latter classified as mowed grasses due to its erect posture and growth to a height above two meters. Some creeping legumes have also been identified, such as Tehuana (*Clirotia ternatea*) and Jarocha (*Pueraria phaseoloides*). In enhanced systems, these plants tend to associate with grasses to provide a mixed forage that is more palatable and, more importantly, has a higher percentage of raw protein and offers greater digestibility. Finally, some varieties of arboreal legumes can be added, since they can be exploited in silvopastoral systems. These include acacias and leucaenas [25,28,30]. Recently, certain improved grasses have become available that should be taken into account when planning efficient pasturing systems, especially in flood-prone zones where buffaloes can be especially productive. The native species that have been improved through methods of selection include Camalote (*Paspalum fasciculatum*), Azuche (*Hymenachne amplexicaulis*) and Aleman (*Echinochloa polystachya*), as well as similar plants that can grow for ample periods in flooded areas but also withstand dry epochs of the year with acceptable production levels, especially when they receive adequate management [19]. This enormous variety of forage species responds differentially in the varied microclimates of the tropics, according to the soil type, humidity, temperature, the incidence of plagues and diseases and other factors [31], but all microclimates support the animal loads in the range of 1–3 AU/hectare. These values turn out to be very competitive when compared to other climates and to systems characterized by low-to-medium levels of the incorporation of technology and capital. This index can be raised by adding improved varieties to well-planned rotating systems to increase the livestock productivity [32].

In most tropical zones, the growth rates of the numerous forage species are associated with the seasonal distribution of precipitation, temperature and annual solar radiation. Forage production normally exceeds the alimentary requirements of cattle during the rainy season when most species reach their peak growth [25]. In contrast, during the dry season (winter and early spring), reduced production and growth rates result in low supply and lower forage quality for free-grazing cattle that predominate in tropical regions [19].

These conditions mean that most producers must develop strategies to optimize the management of these resources. These may involve rotating pasturelands or agrosilvopastoral systems, possibly combined with conservation schemes (silage or haying), moving herds to floodplains and alimentary supplements, among other options [18,32], as strategic responses to the seasonality of forage supplies in terms of both quantity and quality. However, approaches of this kind must be supported by accurate estimates of animal loads and analyses of the optimal periods for occupying pasturelands or letting them rest. The management of these production systems for river buffaloes must accentuate the planned exploitation of pasturelands, since this animal species has shown good adaptation to both the intensive and agrosilvopastoral models thanks to its characteristic gregarious behavior (expressing the herd effect), allonursing (behavior of females who nurse and feed nonfilial offspring) and ease with which it accepts shrub and arboreal (browse) vegetation. This often allows buffaloes to produce yields superior to those of bovines [28,33].

Another important alternative in this regard consists of exploiting native species or introducing species that are especially adaptable to certain environments. The distribution of these species can be planned in accordance with their use; that is, for alimentation, as live fences and/or as shade providers. The latter function is essential for raising buffaloes, especially if only a few ponds are available or if they are absent [33,34]. The practice of establishing trees is growing in the context of rotating pasture systems, but the species contemplated must be able to adapt to a tropical climate and have low-hanging branches that the buffaloes can reach [28,33,35]. In addition to contributing to the welfare and alimentation of these animals, planting trees aids in soil recovery and moisture retention and improve the fertility, while also contributing to the regulation of environmental temperatures [28].

To optimize the exploitation of shrubbery and trees, it is vital to have accurate information on the environment conditions (climate, soils, etc.) to ensure that their biological development and effectiveness as forage fulfillment essential functions in the productive system, especially in year-round pasturing systems, where the goal is to develop a plan for the division and rotation of fields that help raise animal yields. The advantages of this approach to management have been widely documented [9,12,36] and invite participants to emphasize exploring the measures of this kind because of their high functional potential and capacity to increase their productivity throughout the system, especially where bovine cattle are involved [28]. We cannot forget that advancing in this direction requires an integral improvement of production systems that contemplates the reproductive, genetic, sanitary and commercial aspects, among others [37,38].

## 3. Bases and Conditions of the Dual-Purpose System with Buffaloes

As the preceding sections have outlined, a dual-purpose system is one that offers the possibility of producing meat and milk at the same time based on an approach marked by flexibility [39,40] that allows ranches to prioritize either sales of milk (hot, cold or transformed into various dairy products) or meat (as calves, feedlot cattle, half-fattened or fattened animals and even culled individuals) [41]. Decisions in this regard depend on the needs of each rancher’s family, the potentialities of the resources available and the options offered by the market for each product in different tropical areas.

It is under this logic that recent years have witnessed the incorporation of river buffaloes into these productive systems. This species has emerged as an exceptional option for ranchers in tropical zones because of its capacity to adapt to, and develop well in, areas with high temperatures and humidity, to exploit low-quality C4 and C3 plants in flood-prone areas and to move around in heavy soils that may be totally or partially flooded [14,42,43,44]. In these conditions, buffaloes also have the ability to convert abundant vegetation of only a moderate quality into meat and milk products that have high nutritional values. Indeed, buffalo meat and milk have certain properties that can be exploited in differentiated markets where they can attain competitive prices [44,45]. As a result, some of the countries that have recently introduced dual-purpose buffalo production systems [42,46] have restructured and or reorganized the commercial channels to allow consumers to obtain good-quality products while giving ranchers attractive incentives to continue modernizing their productive units.

It is these bio-conditions of large forage supplies—not always of the best quality—that have stimulated ranchers to participate in a long process of developing livestock production systems based on pasturing with reduced alimentary supplementation and adapted to these demanding climatic conditions. These systems have shown gradual improvement in terms of their overall efficiency and benefits for producers, who often assess them as “profitable” [14,47].

The term dual-purpose refers, in essence, to production systems that consist of a set of interdependent elements organized to obtain both milk and meat. The priorities of systems of this kind are to ensure that producers receive adequate incomes and to maintain the economic and environmental sustainability. In this sense, dual-purpose systems contrast markedly to strategies that pursue only profit maximization [41]. In these systems, animals must adapt to conditions that derive from the interaction between the characteristics of their organism and the physical and biotic processes of the environment that surrounds them. This means that the physiology, development and health of the animals are decisive factors in defining the levels of productivity and profitability [32].

**Figure 1:**
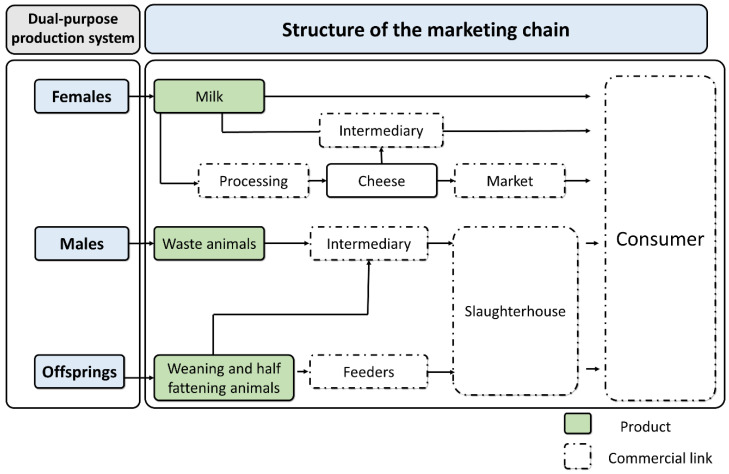
Structure of dual-purpose production systems with river buffaloes.

The locations of ranches; their proximity to markets and their access to forage resources, capital and labor, among other factors, will determine whether an individual system prioritizes milk or meat production in their different modalities [40,41]. However, dual-purpose systems are specifically designed to respond to the factor of the availability of the forage resources. When foraging is abundant, the system gives priority to dairy production, but when it becomes scarce or is of poor quality, meat production will be emphasized over extended periods. In other cases, when foraging is permanently or seasonally scarce, milking may be limited to a certain season, while the meat production may center on selling recently weaned or half-fattened animals (Figure 1). Measures of this type allow livestock farmers to avoid the need to purchase feed products that are often costly and may represent an outflow that they cannot recover when their products are marketed [4].

One typical element of dual-purpose systems that produce bovines is the crossbreeding of Zebu cattle with European animals in an effort to combine, and optimize, environmental resilience with productive potential. In the case of the buffalo, this is less of a conditioning factor, because several of the breeds available—Murrah, Mediterranean, Jafarabadi, Nili-Raví and, in some cases, Carabao—satisfy these requirements individually or through crossbreeding [6,28,30]. In addition, buffaloes offer a great advantage over bovines, because they can thrive in wetlands and floodplains thanks to their capacity to effectively utilize the vegetation that grows there, including species of shrubbery and trees. This ability is found only infrequently among breeds of bovines [11,42,47,48].

Through its emphasis on two products that ranchers can take to market, the dual-purpose approach reduces the impact of economic risks, like price variations, while allowing them to prioritize either milk or meat production in accordance with the environmental conditions (e.g., variations in the availability of milk for offspring), the presence of clients, the contractual conditions offered by markets at a certain time and prices, all of which may lead them to prefer one product over the other at a given moment [4]. Decisions of this kind also operate when events like drought, flooding, plagues, diseases or other eventualities occur, as happens with some frequency in tropical climes, especially under the current conditions of climate change. These phenomena can impact the health, welfare and productivity of herds. In these circumstances, dual-purpose systems offer flexibility while simultaneously mitigating the risks that so often threaten the economic and ecological sustainability of productive units in the tropics [49].

**Figure 2:**
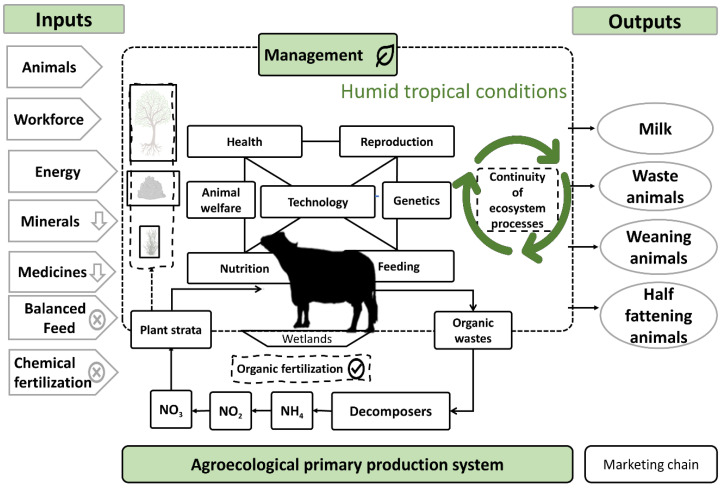
Agroecological conception of dual-purpose production systems with river buffaloes in conditions of the humid tropics.

In synthesis, dual-purpose production systems represent complex assemblages of the processes and practices of synergy and compensation. They can adapt to recurring changes that allow ranchers and their families to develop new arrangements of productive factors or adapt to new approaches in order to achieve their objectives while minimizing their utilization of resources, especially scarce types like capital, while managing the natural regulation of the agroecosystem so as not to perturb the ecosystemic services [50]. Figure 2 presents an image of the complex configuration of the dual-purpose system with buffaloes. The inputs are listed on the left, processes in the middle—closely linked to soil and vegetation—and the products that can be obtained and sent to the market on the right. The figure also illustrates the feedback effects that provide the system with continuity and sustainability [47].

In this context, buffalo production systems require various types of interactions that must be managed as a function of the anatomical and physiological characteristics of this species. In this sense, specialists in veterinary medicine and animal science make contributions of transcendental importance to key areas like reproduction, genetics, nutrition, hygiene and animal welfare and, of course, to topics concerning the safety of the products generated on farms [26]. This view emphasizes the importance of implementing adequate protocols elaborated by specialists to foster the expression of favorable productive and reproductive characteristics related to the economic profitability of the operations [11]. One important aspect to be considered here is the sanitary dimension, since, in some countries in the Americas where buffalos have been recently introduced, insufficient consideration has been given to the fact that these animals are susceptible to disease [51]. Buffaloes are harmed by most diseases and parasites that affect cattle, with the extent to which they are injured varying drastically. Diseases such as brucellosis, tuberculosis, foot-and-mouth disease and, to a lesser degree, leptospirosis, bovine viral diarrhea and fasciolosis have important economic impacts on the water buffalo industry [5,52]. The total or partial absence of the health management protocol could result in serious sanitary and economic problems. Without a doubt, the elaboration and implementation of sanitary programs developed by experts and specialized institutions will aid in mitigating the negative effects of these problems [6,51]. Clearly, the increasing number of buffalo production systems in tropical regions of Latin America requires a greater number of professionals to respond to the demand for specialized veterinary services, not only for activities inside production units but, also, for those that take place outside them, such as analyzing the compositional quality and microbiology of buffalo milk and meat and creating a legal trademark for these products. These aspects are highly significant in terms of guaranteeing the safely of the products generated in these systems [44].

To fully take advantage of the experiences generated by the existing livestock-raising systems, one priority of the dual-purpose approach consists of diversifying activities to include domesticated animals, crops and other vegetable species that complement one another and contribute to producing food and income while also supporting environmental services [18]. However, these systems can also add value to products; for example, in the form of dairy derivatives (that, in the case of buffaloes, are highly coveted). This represents another way to improve the economic performances of these systems, as options of this kind help maintain low levels of inputs, as is traditional in the tropics. Finally, it is in this framework that the river buffalo may represent a viable alternative thanks to its capacity to adapt to, and synergize with, available resources. 

## 4. Towards the Integral Management of a Buffalo-Based System

**Figure 3:**
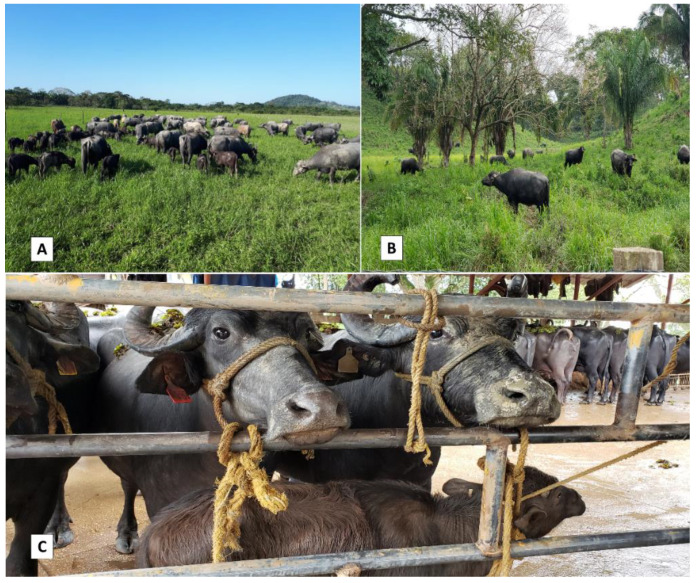
Dual-purpose buffalo production system. (**A**,**B**) The Latin American humid tropics offer abundant forage resources, including shrubs and trees, to which buffalo are optimally adapted. (**C**) Milking with the presence of calves stimulates milk ejection and favors the development of the calves themselves, which is the basis of a dual purpose.

Another characteristic of dual-purpose productive systems that increases their economic viability is that they are directed socially by producers and their families within the confines of individual productive units, though they are affected by actors—individual and collective—that participate through public policies, commercial practices, supply networks and industrialization. It is also important to mention that these systems respond to distinct criteria, including fundamental ones like short- vs. long-term profitability and prioritizing technologies that have a low impact on natural resources [53,54]. This means that the administration of buffalo ranches must take several specific elements into account, including (i) the family management of land, (ii) rational exploitation that conserves sufficient vegetable coverage and foments the persistence of the various strata of vegetation, (iii) the strategic distribution of trees and (iv) preserving sources of water instead of exhausting or contaminating them [35]. It is also advisable to foster an association of grasses and legumes, to divide pasture areas to ensure adequate periods of occupation and rest that favor optimal conditions and, especially, to provide a balanced alimentation for the buffaloes year-round that will be reflected in the outstanding productive indicators. Other key factors include programming adequate intervals between births and indices of parturition and fertility, all of which will contribute to generating higher meat and milk yields (Figure 3) [55].

The availability of the infrastructure and equipment suitable for the objectives of each productive unit is another essential aspect of farms, in terms of guaranteeing the storage and conservation of inputs. Installations should include, for example, shelters for, at least, calves, sick animals and females at the end of gestation. In the case of productive units that include milking operations, it is necessary to provide an adequately conditioned space with suitable equipment and, perhaps, cooling tanks to ensure compliance with the standards of quality and innocuity established for milk and its derivatives that tend to reflect increasing exigencies on the part of clients [40]. Recommendations for production include an inter-parturition interval of 13.8 ± 1.4 months, with 108 ± 7.6 open days, an age at first service of approximately 27.27 ± 1.97 days and first birthing at 37.69 ± 1.96 months, with the goal of achieving a fertility index around 90% at the end of the reproductive period [38,56].

Obviously, the administration of personnel and financial resources are other essential aspects for maintaining the stability of this type of farm [53]. Regarding the personnel, producers must consider ongoing training to familiarize handlers with the processes proper to the river buffalo, which do not necessarily coincide with those of the bovines that workers in these regions are usually most familiar with. In this regard, it is also important to ensure that ponds and shaded areas are available, for these are vital for adequate thermoregulation in buffaloes. Handlers must also be made aware of the fact that this species has specific parturition periods characterized by short days, and that the efficient management of this aspect strongly impacts their reproductive performance, welfare and productivity [10,13,56,57].

Another central issue in the administration of this dual-purpose productive unit involves establishing a recording system that effectively captures data on the productive and financial aspects, such as the number of cells for pasturing, animal loads per hectare and periods of recovery for pasturelands, among other properties of the operation. One especially important element on livestock farms is to control births, fattening periods, lactation, incidences of diseases, levels of weight gain and milk yields, among other key indices [26,43,55]. With respect to financial debits and credits, it is essential to calculate both the unit and total production costs to aid in detecting productive inefficiencies that may generate excessive expenditures in certain items. Towards the end of each production cycle, proprietors should estimate an adequate margin of earnings that allows them to recover their total investment made, including the natural capital, and, of course, provides an adequate standard of living for themselves and their workers [58]. 

Overall, control must be maintained through an agro-ecosystemic vision of the operation that gradually incorporates environmental variables and indicators to begin to estimate both the impact of the operation and possible measures for conserving and restoring natural resources while mitigating the generation of greenhouse gases. Only in this way can operations effectively and integrally advance towards the goal of organizing integral operations that provide for the social welfare of all living beings while also ensuring long-term sustainability.

## 5. Organization of Commercial Channels for Buffalo Products

Fomenting sustainable livestock-raising operations also requires an adequate commercial strategy that offer producers adequate prices to cover both their explicit production costs and those of maintaining ecosystems. Sadly, due to the recent incorporation of tropical areas into the commercial dynamics of many countries, the infrastructure and the means of communication are often not fully developed, though they are necessary for the adequate performance of supply chains, especially for livestock operations that require specific installations and equipment [59,60]. A second concern is that it may be difficult for producers to access mature markets that offer reasonable prices for their products. These obstacles often combine with insufficient levels of organization among producers, the predominant role of commercial middlemen and the scarce regulation of official institutions.

Under these conditions, it is necessary to intervene in the development of markets for buffalo products, especially because meat and milk can be valued as differentiated products. Buffalo meat has low intermuscular fat, caloric, cholesterol and triglyceride contents while providing a greater biological value and iron content compared to beef [44,45]. Buffalo milk has a higher vitamin A content, reduced cholesterol, a lower percentage of water and more dry matter, so its total solids are higher, allowing it to achieve greater yields in dairy products [11,45,61]. Given this, it is important that these products be made available to consumers or, at least, to final distributors, but this requires progress by producers in organizational schemes that will allow them, together, to absorb transaction costs and increase their bargaining power by cementing consolidated sales. Institutions must also participate by supplying necessary public goods, such as markets, warehouses and collection centers, among other infrastructures, and by fomenting strategies and accords among commercial agents that will lead to the development of secure markets or perhaps facilitate encounters between suppliers of buffalo meat and milk products and consumers under conditions of equity and transparency [6].

The experiences of producer organizations are reflected on several well-documented planes, including the establishment of collection centers and, in certain cases, initiatives to transform products into value-added cheeses and sausages through processes that reduce the perishable nature of the original products, allowing more time to negotiate prices and other market conditions [62,63]. This is where producer associations can exert a significant influence on the vertebration of commercial channels to make them better respond to their interests and value their high-quality products and derivatives.

One specific experience that has been widely documented consists in setting up milk collection and distribution centers that make it possible to consolidate supplies, control the quality of the products received and negotiate with clients who are more demanding in terms of volume and compliance with the quality norms [64]. Some centers include warehouses that allow consolidated purchases of inputs like animal feed or medicines and/or laboratories with the minimum equipment required for official quality evaluations that provide objective data for negotiations with clients. The case of meat also presents varied experiences in which producers have been able to establish livestock breed centers, calf auctions and abattoirs that, under their own management, have achieved access to industries and massive consumption centers that allow them to commercialize their entire production at competitive prices [62].

Through these schemes, and with the necessary official support, contractual strategies can be negotiated with clients to cement longer-term sales, design promotional strategies, hire specialized commercial personnel, install packing plants and promote other initiatives that can facilitate the placement of buffalo products so they become increasingly well-known to, and demanded by, consumers, especially those who become convinced of their nutritional qualities [8,44,45].

## 6. Conclusions

Buffalo-raising systems are increasingly earning a profile as a production and commercial option in tropical regions, one that can surpass in several aspects the breeding of conventional species, especially using the dual-purpose model. This model functions because of certain outstanding characteristics of this species, its capacity to adapt to a particularly demanding ecosystem and its capacity to respond to the principles of sustainability in rotating pasture systems, including agrosilvopastoral models. 

Given this logic, buffalo production systems can be adapted to different rationalities, from family-based operations to large-scale enterprises, since they do not entail high degrees of risk or investment but can offer attractive profits, and the capacity to respond favorably focuses on sustainability. However, it is of fundamental importance to continue increasing our knowledge and understanding of the environment and the characteristics of this animal species and to incorporate the capacities of specialists in various strategic areas in order to identify the best management procedures for each component of the production system. Above all, it is important to recognize the specificities of the river buffalo’s physiology and behavior so as to achieve adequate management that focuses on enhancing the animals’ welfare and, therefore, productivity. It is also important to preserve the tradition of tropical pasturing systems, though with processes of planned intensification, such as establishing improved forage species, exploiting the various strata of vegetation and fomenting rotating pasture systems that make it possible to maximize the exploitation of the large supplies of forage that characterize the humid tropics. To the degree that this is accomplished, the exploitation of flood-prone zones and the conservation and restauration of natural resources will attain full viability.

The approach just outlined will permit operations with low production costs but with better yields of higher-quality meat and milk products. This, combined with consolidated forms of producer associations, will progressively open access to more specialized markets that offer remunerative prices. While cattle breeders have important responsibilities in all these initiatives, developments of this kind also require the involvement of other actors, such as distributors, industry, government and consumers. 

## Data Availability

Not applicable.
